# Advances in the Development of Phage-Based Probes for Detection of Bio-Species

**DOI:** 10.3390/bios12010030

**Published:** 2022-01-07

**Authors:** Kameshpandian Paramasivam, Yuanzhao Shen, Jiasheng Yuan, Ibtesam Waheed, Chuanbin Mao, Xin Zhou

**Affiliations:** 1 Institute of Comparative Medicine, College of Veterinary Medicine, Yangzhou University, Yangzhou 225009, China; kameshpandian@gmail.com (K.P.); silenceYZ525@163.com (Y.S.); iamyjs123@163.com (J.Y.); ibtesamwaheed@gmail.com (I.W.); 2 Jiangsu Co-Innovation Center for Prevention and Control of Important Animal Infectious Diseases and Zoonoses, Yangzhou University, Yangzhou 225009, China; 3 Joint International Research Laboratory of Agriculture and Agri-Product Safety, The Ministry of Education of China, Yangzhou University, Yangzhou 225009, China; 4 Stephenson Life Sciences Research Center, Department of Chemistry and Biochemistry, University of Oklahoma, Norman, OK 73019-5300, USA; cbmao@ou.edu

**Keywords:** bacteriophage (phage), biological detection, biosensor, nanomaterials, phage-based probe

## Abstract

Bacteriophages, abbreviated as “phages”, have been developed as emerging nanoprobes for the detection of a wide variety of biological species, such as biomarker molecules and pathogens. Nanosized phages can display a certain length of exogenous peptides of arbitrary sequence or single-chain variable fragments (scFv) of antibodies that specifically bind to the targets of interest, such as animal cells, bacteria, viruses, and protein molecules. Metal nanoparticles generally have unique plasmon resonance effects. Metal nanoparticles such as gold, silver, and magnetism are widely used in the field of visual detection. A phage can be assembled with metal nanoparticles to form an organic–inorganic hybrid probe due to its nanometer-scale size and excellent modifiability. Due to the unique plasmon resonance effect of this composite probe, this technology can be used to visually detect objects of interest under a dark-field microscope. In summary, this review summarizes the recent advances in the development of phage-based probes for ultra-sensitive detection of various bio-species, outlining the advantages and limitations of detection technology of phage-based assays, and highlighting the commonly used editing technologies of phage genomes such as homologous recombination and clustered regularly interspaced palindromic repeats/CRISPR-associated proteins system (CRISPR-Cas). Finally, we discuss the possible scenarios for clinical application of phage-probe-based detection methods.

## 1. Introduction

Bacteriophages, also called phages, are viruses that infect bacteria and are widely distributed on the planet, including in deserts, hot springs, polar waters, sewers, and guts of animals and humans [1,2,3]. They also constitute the most abundant class of viruses on earth, which is believed to outnumber bacteria by several folds [3,4]. Since they were first discovered in 1951, phages have made significant contributions to the development of biology, including their roles in confirming DNA as genetic material and discovering the CRISPR-Cas gene editing system [5]. In recent years, phages have also been used in the treatment of bacterial infections in animals and humans, development of biomaterials for tissue reconstruction, pollution control, and soil remediation [6]. In addition, researchers in the field of biological analysis have employed phages as nanomaterials to develop phage-based probes for ultra-sensitive detection of biological species, such as DNA, protein molecules, viruses, bacteria, fungi, and parasites [7]. This review summarizes the literature on phage-based biological detection, which is divided into four main categories according to different signaling readouts: (1) directly fusing the fluorescent proteins with the phage capsid so that the intensity of the fluorescent signal from phage is proportional to the concentration of targets of interest; (2) directly using phages to infect the host to develop visible plaques or color plaques produced by enzymes reacting with the substrate in the culture medium; (3) constructing a hybrid phage-nanomaterial probe that can specifically capture the targets, with the intensity of the light signal generated by the nanomaterials under natural light proportional to the concentration of the targets; (4) PCR amplification of the specific gene band of phage, with the band intensity corresponding to the number of captured target molecules in the complex. In addition to highlighting the various phage probe-based assays described above, we also introduce two common techniques for phage gene editing, namely homologous recombination and CRISP-cas9, to obtain recombinant phage for construction of the hybrid phage-nanomaterial probes. At the end of this review, we summarize the advantages and limitations of using phage-based probes to detect biological species and look forward to future improvements and application scenarios.

## 2. Fluorescent Signal Generated by Genetically Engineered Phages

**Figure 1:**
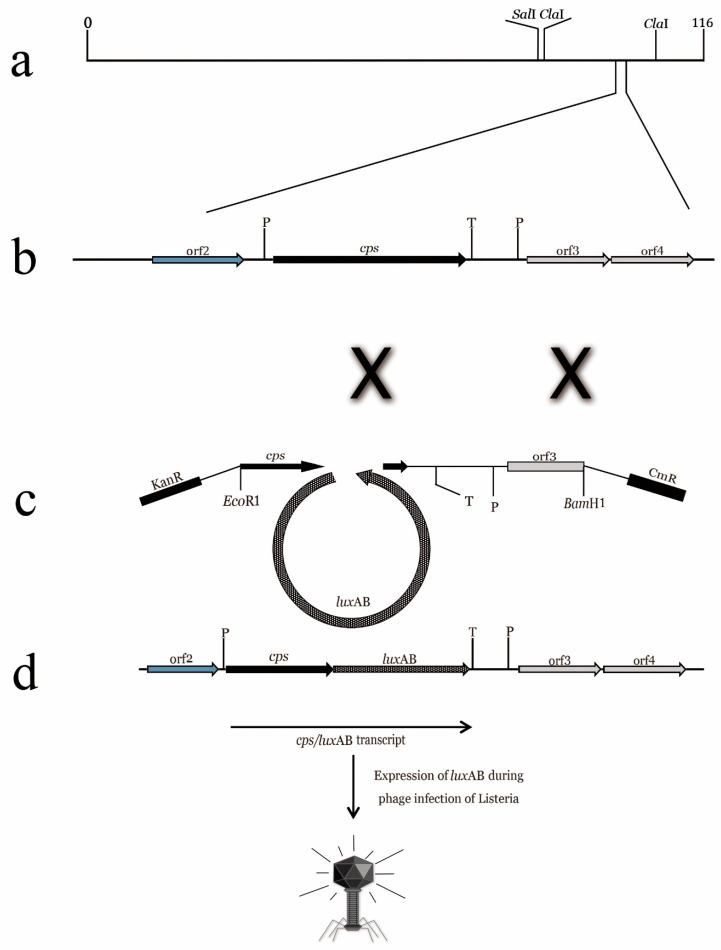
Preparation of “fluorescent phages” by homologous recombination. (**a**) Simplified *Listeria* A511 phage gene map; (**b**) a 5 KB fragment of A511 gene map. Proliferation markers indicate that the phage DNA in infected cells recombined with pcK511, -F3C, and -luxAB fragments (double crossover); (**c**,**d**) luxAB fragment binds between the 3′ end of CPS fragment and the downstream transcriptional terminator. Expression of the A511::luxAB gene produces CPS fragments fused with luciferase genes, resulting in infected listeria cells with bioluminescence phenotype [15].

Phage display technology refers to foreign gene insertion technology that enables a phage to express capsid proteins carrying foreign genes, which was first reported by Smith in 1985 [8]. He displayed a target protein on the surface of a filamentous phage to screen for molecules that could interact with it. Owing to the high throughput and effectiveness of this technique, many scholars have studied and developed a variety of phage display technologies. M13, T4, T7, and λ phage display systems are currently the most commonly used [9,10,11,12]. A phage that displays a fluorescent protein or luciferase and specifically binds to its host bacteria is similar to fluorescently labeled antibodies. For example, as early as 1996, a highly sensitive method for *Listeria* detection was established by phage-specific transfer and bacterial luciferase expression [13]. The luciferase gene was introduced into the phage genome at a certain site using homologous recombination technology, enabling the infected host cells to have a bioluminescent phenotype for identification [14,15,16]. A schematic of the process is shown in Figure 1.

In 2004, Jaye et al. developed a simple yet direct method for labeling M13 phage clones with two common amine-reactive fluorochromes. The pVIII major coat proteins of M13 phages were labeled with fluorochromes and peptides with binding selectivity were displayed on the pIII protein. Positive phage clones and target molecular complexes can be directly observed by fluorescence microscopy or flow cytometry analysis. This method enables multiple fluorescent proteins to label multiple targets, thereby achieving multiplex detection. In addition, the method is capable of multicolor labeling, thereby potentially improving the detection sensitivity of targets with low binding affinity and low abundance, due to the reduction in the number of washing steps [17]. In the same year, Slootweg et al. constructed a recombinant phage plasmid containing a yellow fluorescent protein fused with the GP10 capsid protein. The plasmid was transfected into the host cell BL5403, forming the daughter fluorescent T7 phage for observation under a fluorescence microscope [18]. Using the same principle, Tanji et al. developed a fluorescent T4 phage for the detection of *Escherichia coli* by fusing the small shell (SOC) protein of the T4 phage with green fluorescent protein (GFP) [19]. T4e phages, which do not express lysozyme, were used to avoid the lysis of the *E. coli* host cells. Positive clones of GFP-labeled T4e-phage in host cells can be distinguished by their green fluorescence. The specific capsid protein of the pathogen, and peptides binding the antigen can be obtained by screening a random peptide phage library. Thereafter, the fluorescence protein is displayed on the other capsid proteins of the phage. Therefore, the bi-functionalized phage carrying both the binding peptide and a fluorescence protein can be used as a candidate probe for detection of the pathogen by ELISA, phage dot hybridization, and immunoprecipitation analysis [20].

Fluorescence phage-based detection methods are considered to be a promising method and are expected to become a substitute for current conventional detection techniques [21] (Schofield et al., 2012). Phages theoretically have the ability to display multiple types of peptides on the surface [22] (Lee et al., 2017), and the protein or peptide–protein interactions are basically the most important evolutionary driving force in life phenomena. Therefore, the recombinant phages that have been screened have the potential to act on target proteins with high specificity, recombinant phage particles are directly used in Immune PCR as the reagent, and the single-chain variable fragments (scFv) displayed on the surface and the phage DNA itself can be directly used as detection antibodies and PCR templates, respectively. Hasmoni et al. introduced a phage enzyme-linked immunosorbent (phage-ELISA) assay to detect the hepatitis B virus. The phage-ELISA is a modified ELISA-based technique with the primary antibody replaced with the fusion phage. To verify the sensitivity of this assay, a fusion M13 phage that interacts with HBcAg (the core protein of nucleocapsids of HBV) was isolated from a phage display cyclic peptide library. The serum samples with HBcAg were coated onto a 96-well plate, then incubated with the fusion phages and anti-M13 monoclonal antibody conjugated with HRP, successively. Finally, the absorbance at 405 nm was determined in a microtiter plate reader. In this study, the LOD of phage-ELISA assay was about 10 ng with 1.0 × 10^12^ pfu/mL of the purified fusion phage [23].

Although the M13 fluorophore system is highly selective, these complexes typically have poor molecular detection limitations due to their low absorption cross sections and moderate quantum yields. To overcome these challenges, Huang et al. reported a novel method. In this study, co-assembly of the M13 virus, cyanine 3 dye, and silver nanoparticles was developed to create fluorescent markers capable of combining molecular precision and high emissivity. The enhanced emission of cyanine 3 up to 24 times can be achieved by varying the size of nanoparticles and particle fluorophore separation. In addition, fluorescence enhancement was found to increase with the increase in dye surface density on the viral capsid. Finally, the high-fluorescence probe was used for in vitro staining of *E. coli*. These results demonstrate an inexpensive framework for achieving tuned fluorescence enhancement [24].

**Figure 2:**
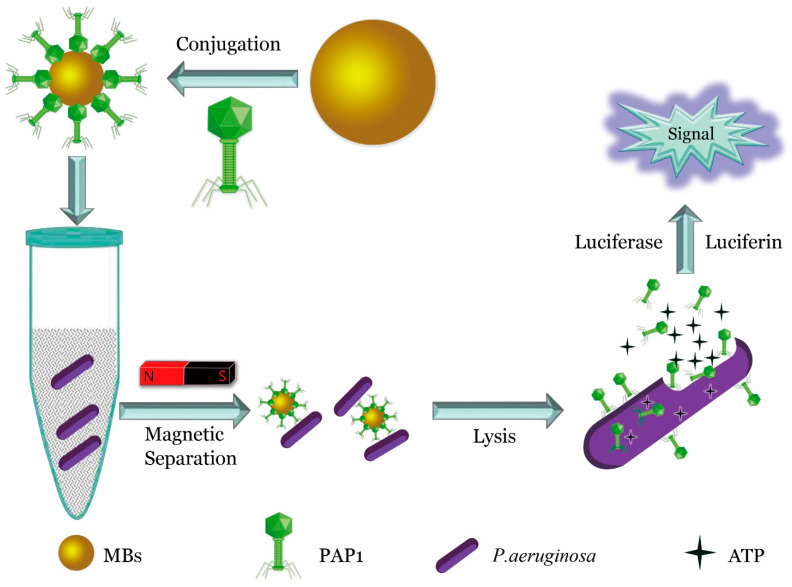
Quantitative detection of *P. aeruginosa* using specific phage. Highly specific fusion phage and magnetic bead assembly to construct a specific probe for enrichment of *P. aeruginosa*, and release of ATP from the bacteria through phage lysis. ATP was quantified to determine the presence and quantity of *P. aeruginosa* [25].

Even natural, unmodified phages can be widely used if combined with appropriate innovative detection platforms. For example, in 2017, He et al. isolated a virulent phage strain, PAP1, with high specificity to *Pseudomonas aeruginosa* (*P. aeruginosa*) from sewage. By combining PAP1 with magnetic beads, they constructed a magnetic PAP1 probe for the enrichment and detection of *P. aeruginosa*. The magnetic PAP1 probes were first used to capture *P. aeruginosa* and then infect and lyse the host to release ATP. The number of *P. aeruginosa* was determined using a firefly luciferase-ATP bioluminescence system [25]. The detection schematic is shown in Figure 2.

**Figure 3:**
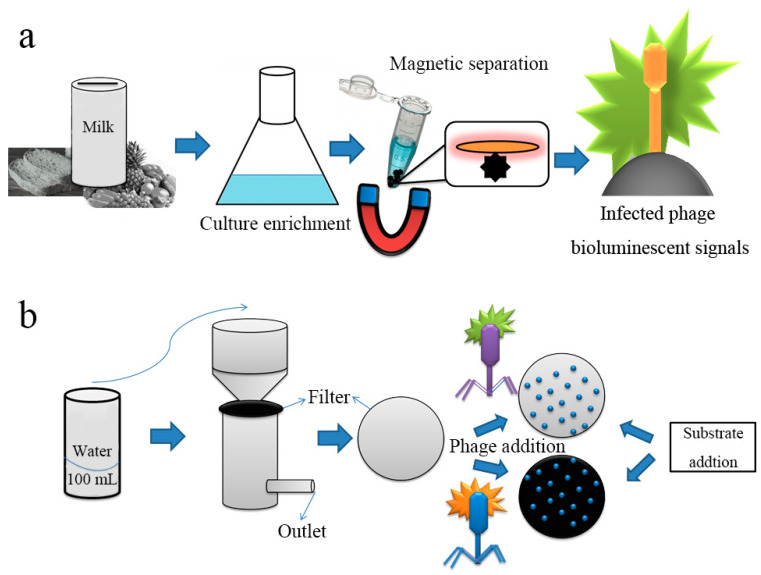
Schematic representation of phage-based bioluminescent detection. (**a**) Pathogens in the contaminated food sample were detected by CBD-MS based genetically engineered phage with readout of bioluminescence signal; (**b**) pathogen in contaminated water detected by filter medium based recombinant phage infection and protein expression on substrate addition [26,27].

The combination of biology and chemistry with material science has helped develop phages as powerful research tools for nanomedicine. Future developments in phage diagnostic technology will require the cooperation of scientists from many fields and is expected to entail unique methods and strategies. Some important issues remain to be resolved, and the development of phage nanomaterials is still limited to a few monotonic phage structures; thus, the discovery of new phages with new properties will help to address these issues. In 2018, the Loessner research team described a diagnostic protocol for *Listeria* using cell wall-binding domains of *Listeria* phage endolysins-based magnetic separation (CBD-MS), A511::luxAB phage infection, and generation of bioluminescent signals [26]. This combined method has high sensitivity with a limit of detection (LOD) of 0.1 to 1.0 cfu/g and an assay time of less than 22 h (Figure 3a). In the same year, Nugen’s team developed a phage-based membrane filtration assay for the detection of *E. coli* in drinking water using genetically engineered T7 phages [27]. Both luciferase and alkaline phosphatase were fused to the genes of the T7 phage using carbohydrate-binding modules specific to cellulose. The binding modules facilitated the immobilization of the reporter probes on the cellulose filter in proximity to the lysed cells. After mixing the recombinant T7 probes with *E. coli* in drinking water, T7 phages specifically infected *E. coli*. Following substrate addition for the alkaline phosphatase and luciferase reporters, the enzymes were expressed and released from the bacterial cells during the infection. Consequently, the bioluminescence signals were monitored by the naked eye or by bioluminescence imaging (Figure 3b).

In recently, Chen et al. used a genetically engineered reporter T7 phage to detect a food-borne pathogen *E. coli* via fluorometric detection [28]. This study suggests that a fluorogenic substrate to detect β-gal activity caused by the genetically modified phage infection is a unique and very promising way to detect bacterial pathogens.

## 3. Formation of Visible Monoclonal Plaques

**Figure 4:**
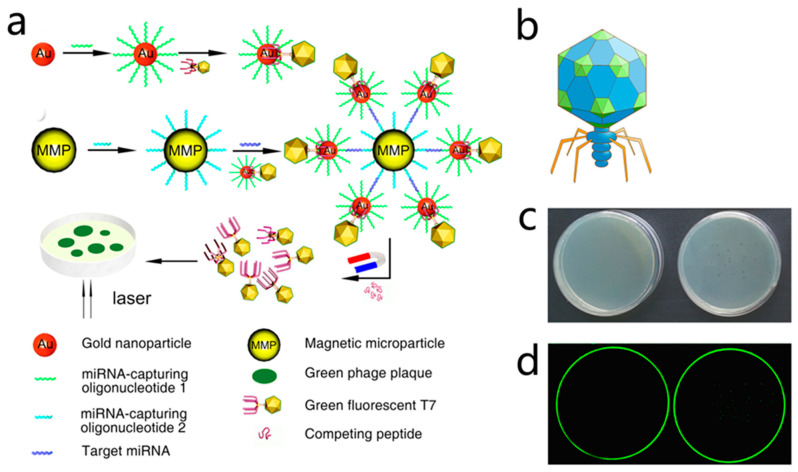
Phage-mediated ultra-sensitive visual detection of miRNA. (**a**) The oligonucleotides that captured miRNA were first coupled to GNP. This was then coupled to a fluorescent T7 phage containing a gold-binding peptide (GBP) on its tail fiber to prepare the T7-GNP probe, with specific one-to-one binding of T7-GBP to GNP. An MMP probe was prepared by binding another biotinylated miRNA capture oligonucleotide to the MMP. Then the MMP probe and the T7-GNP probe were mixed with the target miRNA. GNP and MMP probes trapping the target miRNA resulted in the formation of sandwich complexes. These two different miRNA capture oligonucleotides are complementary, and thus bind to two different segments of the same miRNA target molecule (at the two distal ends), facilitating the formation of sandwich complexes. When high concentrations of GBP complexes competing for GNP binding sites with the T7 phage were added, the T7 phage was released from the complex. The T7 phage was then magnetically separated. Plaque was formed by spreading purified T7 phage in a culture dish. Since the number of fluorescent plaques was equal to the number of T7 phages, which was equal to that of target miRNA molecules, the amount of miRNA could be determined by calculating the number of fluorescent plaques. (**b**) Schematic diagram of recombinant fluorescent T7 phage and the GFP on the envelope of recombinant fluorescent T7 phage; (**c**) petri dish photograph showing the transparent plaque visible to the naked eye after the green fluorescent T7 phage was cultured on the host bacterial culture medium (right). When no phage infected the bacteria, there were no plaques in the culture dish (left). (**d**) In a fluorescent scanner, the image of the same Petri dish shown in 488 nm excited light scan shows a green patch on the right and no green patch on the left [36].

As early as 1938, phages have been used to classify bacteria by phage typing. Phage typing takes advantage of the differences in susceptibility of bacteria to various phages, allowing identification of the genera and species [29]. This method has been applied to a variety of bacteria based on the detection of plaques on phage replication and lysis of bacterial cells. In 2008, Kafatos et al. evaluated the application of phage typing as a *Salmonella* surveillance system. Phage typing yielded better specificity than traditional methods, which are reported to miss 40% of the epidemic cases [30]. However, there are many limitations in using phage typing as a basis for diagnosis. For example, phage typing relies on the rate of plaque formation (at which the phage infects and lyses the host bacteria), which is commonly time consuming, especially for slow-growing bacteria such as mycobacteria. To develop faster and more economical detection methods, some scholars have combined the characteristic of phages to inhibit host growth with other sensitive technologies to obtain an updated version of the pathogen detection technology. For example, He et al. combined phage *D29* that specifically infected *Mycobacterium tuberculosis* with a multichannel series quartz crystal sensor (MSPQC) to develop a phage-amplified MSPQC assay, which is faster and more economical than the BACTEC960 MGIT method used in clinical *M. tuberculosis* detection [31]. To improve the efficiency and sensitivity of phage detection technology, Nugenet al. from Cornell University designed a T7 phage containing the lacZ gene, which induced β-Gal over expression in the phage infection amplification cycle by the T7 promoter, and improved the sensitivity of detection of *E. coli* cells by chemical fluorescence method [32]. With the advent of these methods, many laboratories have begun to study a variety of specific host detection methods based on phage amplification [33,34,35]. Because these detection methods combine biology, chemistry, and nanotechnology, they have many advantages, such as efficiency, high sensitivity, strong specificity, and low cost. In 2015, Zhou et al. reported a naked eye counting of miRNA using a T7@GNP probe, which was prepared by assembling a T7 phage with gold nanoparticle (GNP) in a “one-to-one” manner. This strategy demonstrated a traceable count of a single molecule with the naked eye [36], which is a challenging project in molecular diagnosis. They displayed a fluorescent protein on the surface of the capsid protein and a gold affinity peptide with the tail filament protein, thereby producing a T7@GNP probe. Meanwhile, a magnetic microparticle (MMP) probe modified with DNA sequences complementary to the other end of the target miRNA was prepared. Therefore, if the target miRNA exists in the sample, the T7@GNP and MMP probes can capture the target miRNA and form a sandwich structure of MMP + miRNA + T7@GNP. In the sandwich structure, the number of phages was the same as that of the target miRNAs. After the sandwich structures were separated by a magnetic force, the phages were released from the sandwich structures by adding a competitive agent or pure water, and the released phages were collected and plated on the host bacterial culture to develop the plaques. Because a phage can form a visible plaque on the host plate (fluorescent plaque under a fluorescence scanner) and the number of plaques can be counted directly with the naked eye, the number of captured target miRNA molecules can be counted (Figure 4).

**Figure 5:**
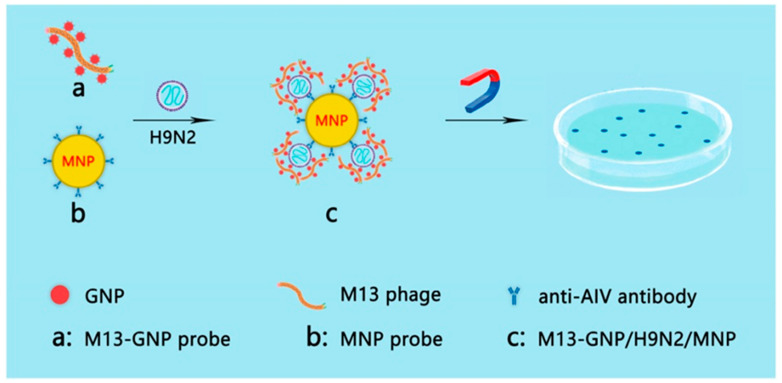
Strategy of counting viruses with the naked eye. (**a**) M13@GNP probes were prepared by assembling M13 phage with GNPs modified with antibodies against M13 p8 and with antibodies against H9N2 virus; (**b**) magnetic nanoparticles (MNP) probes were prepared by mixing MNPs with the monoclonal antibodies against H9N2 virus; (**c**) sandwich complex formed by H9N2 viruses captured by both M13@GNP and MNP probes. The M13 phages were separated from the complex and plated to infect host *E. coli* ER2738 on LB agarose plate with IPTG/Xgal. The blue plaques indicate the presence of H9N2 virus [38].

Based on this method, Peng et al. developed a colorimetric method based on the conjunction of GNP and M13 phage. This method can detect *Escherichia coli* with the naked eye. This method can also detect ~100 cells with no cross-reactivity [37]. In 2021, to achieve direct inspection of targets of interest with the naked eye rather than using fluorescent scanners, an M13 phage-GNP probe was constructed by assembling GNPs modified with both monoclonal antibodies for the p8 protein of M13 phage and those for hemagglutinin of theH9N2 virus with the nonlytic M13 phages [38]. In addition, magnetic nanoparticle (MNP) probes were prepared by coupling the H9N2 monoclonal antibodies with MNPs. H9N2 viruses in the sample were first captured by MNP probes and then co-captured with M13@GNP probes to form sandwich complexes. After separation with magnetic power and release with dilution solution, M13 phages were cocultured with the host strain on the LB agar plate containing IPTG/X-Gal and developed blue plaques, the number of which has a strict quantitative relationship with that of viruses, reaching 50 PFU/mL. This visible counting method is more intuitive and sensitive than the traditional qPCR method (Figure 5).

## 4. Signals from Nanomaterials Conjugated with Phages

**Figure 6:**
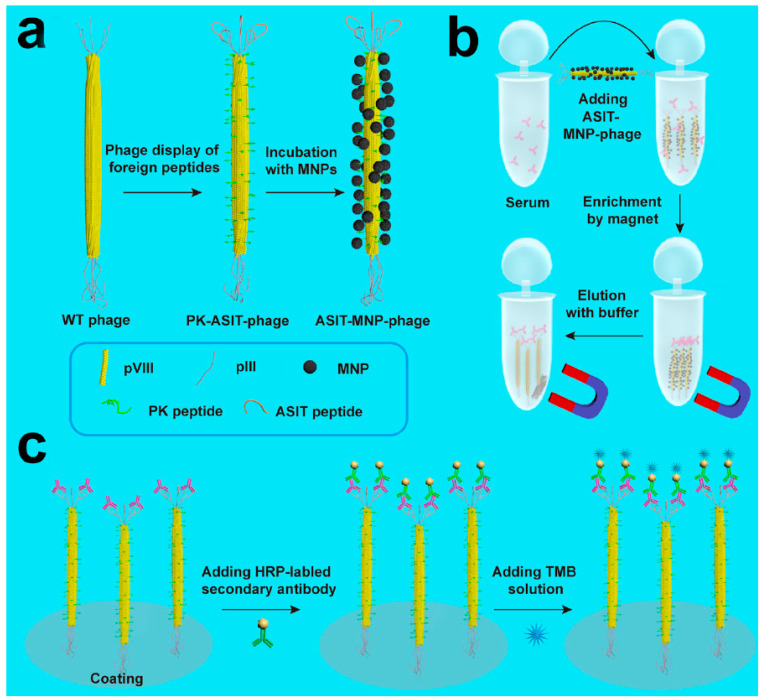
The “Nanofiber” capture antibody detection strategy. (**a**) The peptides “PK”(MNP-binding) and “ASIT” (anti-Sap2-IgG-binding) are displayed on the pVIII protein (the major envelope protein on the side wall) and the pIII protein (top secondary envelope protein) of the wild-type phage, respectively. Then, MNPs bind to the MNP-binding peptide, forming the ASIT–MNP–phage complex. (**b**) ASIT-MNP-phages were added to human serum to capture biomarkers through its pIII tip (anti-Sap2-IgG), and were enriched with a magnet, followed by elution. (**c**) The eluted ASIT-phage/biomarker complex was then coated on the ELISA plate, and Horseradish peroxidase (HRP)-labeled bis-antibody and TMB staining solution were added to the synthesized complex to form a chromogenic reagent for detecting biomarkers. PK, MNP-binding peptide (PTYSLVPRLATQPFK); ASIT, anti-Sap2-IgG targeted peptide (VKYTS) [43].

Phage-based plaque detection has the advantage of high sensitivity, but it is time consuming. With the advent of chemical biology, a type of organic–inorganic hybrid probe formed by phage coupling to nanomaterials has been developed, based on the chemical modification of phages and the assembly of metal nanoparticles with the modified phages [39]. The optical properties of nanomaterials can be used for rapid and sensitive detection. For example, some metal nanomaterials (silver or gold) have strong plasmon resonance effects, so they can directly reflect the presence and quantity of the objects detected in samples once the hybrid probes capture and form complexes with the targets of interest [40]. To prepare such hybrid probes, two issues must be addressed. First, phages need to be genetically engineered to have a high affinity for the targets of interest. Second, phages need to be chemically or genetically modified to form strong bonds with the nanomaterials. Several successful studies have been published in this regard. For instance, in 2006, Edger et al. reported a sensitive, rapid, and simple method for the detection of *E. coli* bacteria by combining in vivo biotinylation of engineered host-specific T7 bacteriophages and conjugation of the phage to streptavidin-coated QDs. This method utilizes the strong fluorescent nature of quantum dots under light to achieve highly sensitive (10 bacterial cells/mL) and rapid detection (within 45 min) of bacteria [41]. Wang et al. reported a phage-based magnetoelastic (ME) biosensor method for the detection of *Salmonella typhimurium* from fresh spinach leaves. Filamentous E2 phage was immobilized on a gold-coated strip, then incubated the *Salmonella typhimurium* sample on the surface of biosensor. Finally, the change of resonant frequency on the biosensor surface was used to determine the *Salmonella* infection, the LOD of this method can reach to 4 cfu/g [42]. Furthermore, if host-specific phages can be coupled with different emission color quantum dots, they can be used for the simultaneous detection of different strains in the same sample. Another example of bacteriophage metal nanoprobes relates to the detection of *Candida albicans*, which is an important pathogen in clinical practice and can cause high mortality in patients with cancer. Before the advent of phage-mediated detection, blood culture was the gold standard for the clinical diagnosis of *C. albicans* infection, which required five days to determine whether a person is infected with *C. albicans*, leading to delayed treatment. Mao et al. in 2015 developed a “nanofiber assay”, which is capable of quick and sensitive detection of antibodies against *C. albicans* in human serum [43]. Magnetic nanofibers were first prepared with genetically engineered M13 phages using magnetic beads, and that M13 phage expressed surface-specific peptides against *C. albicans*. These nanofibers were added to the serum sample to enrich the target protein, and then the complexes were harvested with magnetic power. Finally, the phages were eluted from the complexes and assayed by ELISA. By displaying a polypeptide that binds specifically to pathogens, phages can bind magnetic particles to form a magnetic phage, thus achieving rapid separation. A schematic of this process is shown in Figure 6.

**Figure 7:**
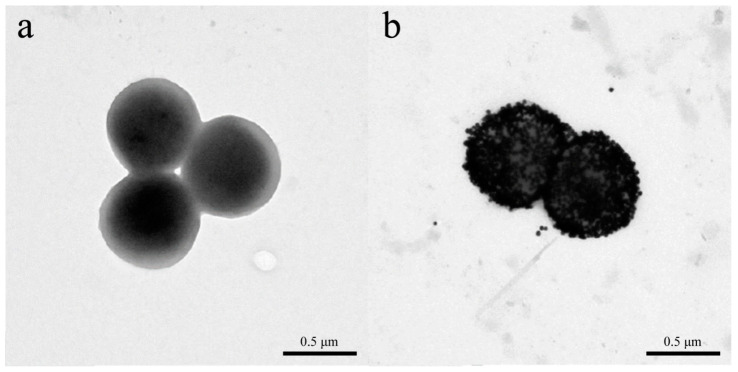
Transmission electron microscopic characterization of nanophage probe binding to *Staphylococcus aureus*. (**a**) *S. aureus* cells. (**b**) AuNP probe-*S. aureus* conjugates [44].

In 2016, Liu et al. reported a colorimetric biosensor for *Staphylococcus aureus* detection using a GNP probe modified with a CS-AuNP@pVIII fusion protein and a phage monoclone with GQTTLTTS obtained by screening with a f8/8 landscape phage library can specifically bind *Staphylococcus aureus* [44]. The modified probes exhibited good affinity for *Staphylococcus aureus* as demonstrated by transmission electron microscopy, which showed that thousands of probes bind to the surface of the pathogen (Figure 7). This method is similar to the widely used colloidal gold test strip, which correlates the color intensity of Au nanoparticles with the concentration of the target of interest. The rapid colorimetric method can detect *S. aureus* in 30 min and has ultra-high sensitivity, with an LOD of 19 cfu/mL. For micrometer-scale pathogens, such as parasites or bacteria, thousands of GNP bind to the target, thus making a single pathogen visible to the naked eye under a dark-field microscope. However, for molecules or small virus particles, which use the strong surface plasmon resonance (SPR) effect generated by clumps of nanomaterials, visual detection is not possible, as it is possible only in the presence of millions of targets of interest in the reaction.

In addition to detecting proteins and microbes, phage-based probes can also be used to detect metal ions. For instance, Wang et al. reported a network-like phage@GNPprobe as a biosensor for the detection of mercury [45]. Dispersion forces between closed-shell metal atoms are highly specific and strong, and are greatly magnified by relativistic effects, particularly when these interactions involve heavy ions such as Hg^2+^ and Au^+^ [46]. The Hg^2+^-binding M13 phage formed networks with AuNPs, and subsequent reduction of mercury to Hg (0) led to its deposition on the AuNP surfaces, causing a blue shift in the SPR of AuNPs and increasing the absorbance. An LOD of 8 × 10^−8^ mol L^−1^ was achieved by measuring the absorbance of AuNPs at A525/A650. The affinity of bio-panned M13 phage binding with Hg^2+^ and that of AuNPs—Hg^2+^ binding showed that high selectivity and high interference tolerance capability results in high sensing performance.

In 2018, the “one step” production of M13 engineered phage-bio-functionalized silicon nanoparticles specifically bound the Peripheral Blood Mononuclear Cell [47]. The PLAL has potential use for in vitro application and as an optical biosensor used to detect a specific target. This is low cost, easy to prepare by one step and has high yield, which is very useful to biomedical application. Similarly, one-step synthesis of M13 phage-based nanoparticles has fluorescence properties [48]. This report indicates using virus particles as a carbon source for fluorescence nanoparticles synthesis. In 2019, early Alzheimer’s disease was detected using a phage display screening method [49]. This kind of detection will help as to develop more blood biomarkers for early diagnosis of Alzheimer disease. In 2020, Peng and Chen reported a chimeric phage nanoparticles for rapid characterization of bacterial pathogens [50]. The thiolated M13 phage binds AuNPs and its transformation into visible color can be detected calorimetrically. This report gives the bacterial species identification in a short period. The real-time Single-Walled Carbon Nano Tubes (SWNT) coupled with genetically modified M13 Phage based fluorescence imaging help in ovarian cancer debulking surgery [51]. This report suggests that SWNT-Phage fluorescence imaging improves the real-time identification and resection of microscopic tumors, and increases the post-operative survival. Recently, Fiskin et al. developed a PHAGE-ATAC (Assay for Transposase-Accessible Chromatin) method that uses phage display. Engineered nanobodies (single-domain antibody) measure the single cells protein and chromatin accessibility profile as well as mtDNA-based clonal tracing [52]. The PHAGE-ATAC was used in multimodal analysis of human immune cells, intracellular protein and detection of SARS-CoV-2 spike protein in human cell population. In future, this synthetic, a complex and large phage library could enhance multimodal, single-cell characterization of protein, epigenome, and other profiles with high specificity.

## 5. Phage-Based PCR for the Detection of Target of Interest

**Figure 8:**
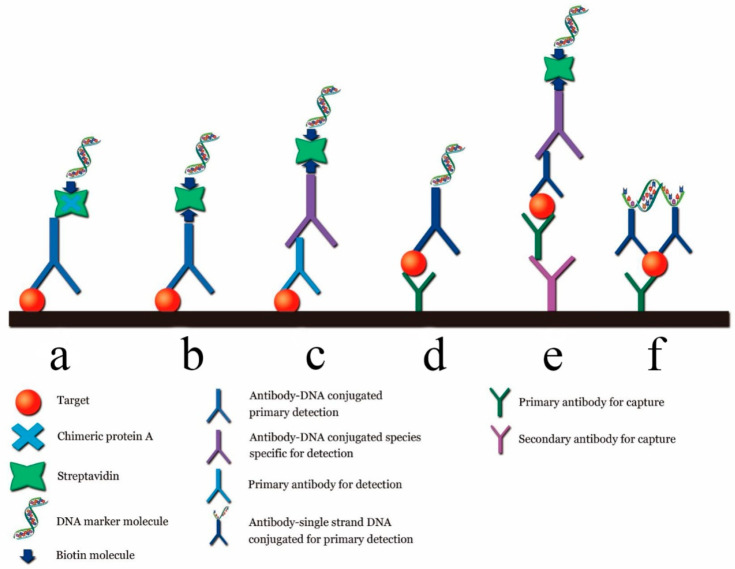
Different immune PCR strategies. (**a**) Original immuno-PCR; (**b**) universal direct immuno-PCR; (**c**) universal indirect immuno-PCR; (**d**) direct sandwich immuno-PCR; (**e**) indirect sandwich immuno-PCR; (**f**) immuno-PCR by proximity ligation [55].

In the early stage of tumors, the content of biomarker molecules in the serum or tissue is generally very low [53]. Therefore, noninvasive liquid detection requires highly sensitive detection technologies. Ultra-sensitive protein detection techniques can be used to intervene early in the disease, slow its progression, or cure it. However, the current clinical protein detection technology is considerably less sensitive than PCR and cannot meet the requirements of early noninvasive detection [54]. The LOD of the most widely used ELISA kit for biomarker proteins is approximately 0.1~1.0 ng/mL under different testing conditions [55]. However, PCR can be combined with the immunoassays to develop an immune-PCR technique, the sensitivity of which can be increased by two to three orders of magnitude compared with that of ELISA [56]. Immuno-PCR techniques typically employ the Fc terminal of an antibody coupled with streptavidin, followed by its combination with DNA with a biotin-modified end. Schematic diagrams of general immune-PCR strategies for the detection of antigens are shown in Figure 8.

In 1992, Sano et al. first introduced a PCR-based immunoassay method [57], where they used PCR to amplify the reporter gene to a diagnostic concentration after the immune reaction and established an immuno-PCR detection method to detect low-abundance proteins. The biotinylated DNA was specifically connected to the antigen–monoclonal antibody complex immobilized on the wells of the microtiter plate, then PCR amplification was performed. Finally the PCR products were analyzed via agarose gel electrophoresis. The LOD of PCR-based immunoassay method can easily reach to 580 antigen molecules (9.6 × 10^−22^ moles). In 2021, Rizoo et al. established an engineered phages assisted real-time qPCR method for *Escherichia coli*, *Pseudomonas aeruginosa*, and *Staphylococcus aureus* detection. M13 phage was modified with specific peptides to capture bacteria, then the complex was directly quantified with qPCR method on a silicon microchip. The LOD of this method can reach to 10 cells/reaction [58]. Immuno-PCR (IPCR) is a powerful detection technique in immunology research and clinical diagnosis owing to its hypersensitivity. The initial IPCR method typically used streptavidin chimeras, which are combined with biotinylated linear plasmids to detect antibodies and report DNA [59]. Subsequently, a novel self-assembly nanostructure network based on biotin-streptavidin interactions was proposed and has been widely used in basic immunology and applied immunology research [60,61]. However, the preparation of chimeras and nanostructure networks is difficult and complex, which hinders the widespread application of immune-PCR. In order to construct a simple and universal reporting system for immuno-PCR detection, some researchers in 2006 proposed the use of natural phage nanoparticles for immuno-PCR detection. *Hantavirus* nucleocapsid protein and prion protein were used as targets for phage display-mediated immune amplification for detection [62,63]. The recombinant phage with a single-chain variable fragment (scFv) specifically targeted the NP or PrP antigen. The scFv was bound to DNA by connecting molecules, and then amplified by PCR, so that the sensitivity of quantitative detection of antigen was excellent [64]. In addition, to further reduce the number of false positives, magnetic beads were added to purify the target antigens after the formation of phage–antigen complexes. The specificity of the detection was further improved using this two-step capture strategy [65,66].

**Figure 9:**
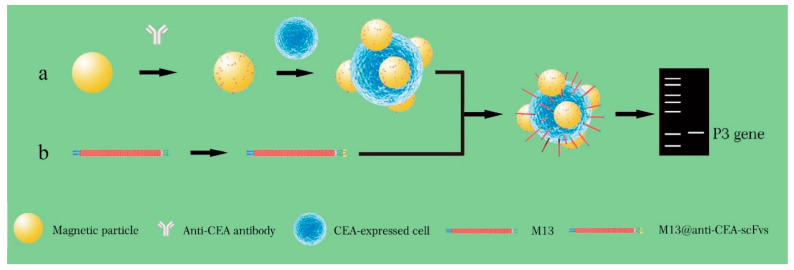
Detection of CEA-expressed cells using M13KO7-ACEA scFv phage (Cited from 62). (**a**) Magnetic nanoparticle (MNP) probes were prepared by mixing MNPs with anti-CEA (carcinoembryonic antigen) antibodies, followed by separation with magnetic power and washing three times. The MNP probes were first used for pulling down CEA overexpressed CTC in the blood sample and subjected to binding by M13 probes to form the sandwich complexes. (**b**) The M13 phage with p3 displaying anti-CEA scFv was constructed using the pCANTAB-5E-anti-CEA scFv plasmid. The plasmids were transformed into *E. coli* TG1 cells and then the cells were infected with M13KO7 helper phages. After 2 h, the recombinant M13@anti-CE-scFvs were purified from the *E. coli* TG1 culture supernatant. M13@anti-CE-scFv phages can specifically bind to CEA antigens, which were overexpressed in colorectal cancer cells. M13@anti-CE-scFv phages were subsequently added into the MNP@CTC and formed the MNP@CTC@M13 anti-CEA-scFv complexes. After magnetic separation, the MNP@CTC@M13 anti-CEA-scFv complexes were detected by PCR with p3 gene as the template. M13 phage-based PCR can rapidly detect single CTC in a small volume of blood sample.

In addition, Zhou’s team recently demonstrated a phage-based detection strategy for the identification of a single circulating tumor cell (CTC) [67]. The key assay component is the flexible recombinant M13@anti-CEA-scFv probe (M13 and CEA stand for M13 phage and carcinoembryonic antigen, respectively), which has a high affinity for CEA antigen expressed on the surface of intestinal cancer cells, as confirmed by ELISA. Cell immunofluorescence experiments by confocal microscopy also demonstrated that M13@anti-CEA-scFv could specifically bind to CEA-positive colon cancer cells such as Caco-2 and HT29, instead of CEA-negative HEK293T cells, even when negative cells were in great excess. qPCR further proved the ability to pin down single Caco-2 cells in 1 mL of spiked samples by 1000 copies of M13@anti-CEA-scFv probe after merely 15 min of incubation. Another probe comprising a rigid magnetic microparticle (MNP) loaded with anti-CEA antibody was used for preliminary screening of the single CTC. The CEA on the resultant MMP/CTC complex was subsequently recognized by numerous copies of M13@anti-CEA-scFv to form the MMP/CTC/M13 @anti-CEA-scFv sandwich complex. Probing phage DNA in this complex by PCR leads to the unambiguous identification of a single Caco-2 cell in 1 mL of spiked blood samples (Figure 9).

Recently, the Peiwu Li research team developed a phage display-mediated immune polymerase chain reaction (PD-IPCR) method for the simultaneous quantitative detection of total aflatoxins and zearalenone in cereals [68]. In this study, mAb 1C11 (anti-aflatoxins monoclonal antibody) and mAb 2D3 (anti-zearalenone monoclonal antibody) were modified in 96-well polystyrene microplates. V_2–5_ and V_8#_ phages displaying the variable domain of the heavy chain anti-idiotypic nanobody that binds 1C11 or 2D3 were used as competitors for the corresponding analytes. Specific DNA sequences encoding the anti-idiotypic nanobodies were used to design primers for real-time PCR amplification detection. The LOD values for total aflatoxins and zearalenone in a sample were 0.03 and 0.09 ng mL^−1^, respectively. Similarly, Nzuma et al. reported an immunomagnetic separation-quantitative PCR (IMS-qPCR) method to detect low numbers of *Campylobacter jejuni* using recombinant scFv antibody from phage display. To specifically capture *Campylobacter jejuni*, the surface of the magnetic beads was modified with a large number of monoclonal antibodies, thereby specifically binding to the target in a complex environment, and then a magnetic field was applied to the entire reaction system to separate the target sample from other interference items [69]. The scFv antibody phage-display library was screened by surface bio-panning against *C. jejuni* cells. The enriched clones were analyzed by ELISA. Two scFv antibodies had strong and specific recognition towards the *C. jejuni* cell. In Western blot analysis one antibody, scFv80, was expressed as a soluble protein and retained its specific and strong binding against *C. jejuni* cells. This recombinant monoclonal scFv antibody and the covalently coat paramagnetic beads were used for IMS-qPCR. The “gold standard” culture-based technique for *C. jejuni* detection typically takes 3–5 days, but IMS-qPCR can achieve sensitive detection within 3 h. This phage-mediated immune-PCR method, which combines the specificity of phage display antibody with the sensitivity of PCR, is expected to be widely used for rapid and ultra-sensitive analysis of clinically specific samples. For example, a novel method PAA-qPCR (phage amplification combines with qPCR) was reported to detect *Salmonella enteritidis* in chicken meat samples [70]. Similarly, *Acinetobacter baumannii* has recently been detected using phage-based qPCR method [71].

## 6. Techniques for Editing Phage Genome

### 6.1. Homologous Recombination Technique for Editing of Phage Genome

Homologous recombination was the earliest technique used for phage genome modification [72]. Two identical or similar phenotypical parental phage genomes were exchanged based on homologous or similar DNA segments in the host bacterium, and the progeny were screened based on specific phenotypic characteristics. Due to the lack of site specificity, homologous sequences carried by the plasmids are integrated into the phage genome through homologous recombination to obtain the modified phage [73,74]. First, the desired genome was mutated and inserted along with phage homologous DNA into the selected plasmid using standard restriction digestion and ligation, PCR, and site-directed mutagenesis. These genetically modified plasmids were then transformed into host bacteria, which were then injected with phages. The genetically modified phages were screened, but the recombinant phage concentration obtained was very low and the method was time consuming [75,76]. Next, based on efficient recombination engineering and electroporation technology, two main methods were developed for lytic phage recombination: a lambda phage model system and a *Mycobacterium* phage model system [77,78,79,80,81]. In the lambda phage engineering system, the homologous recombination steps include phage infection, induction of recombination function, competent cell preparation, induction of dsDNA or ssDNA substrate by electroporation, recovery of phage plaques after lysis cycle, and phage mutation analysis. The *Mycobacterium* recombination engineering system uses a new and potentially powerful method called phage recombineering of electroporated DNA (PRED). Phage recombination has been proven to be an efficient method to construct gene deletions, small fragment insertions, gene replacement, and point mutations in lytic phages, though the recombination rate still needs to be improved. Homologous recombination technology has a wide application prospect; in 2021, Erickson et al. isolated a novel bacteriophage named L. grayi bacteriophage (LPJP1). LPJP1 is the first and only reported jumbo bacteriophage infecting the Listeria genus. This team used homologous recombination technology to reprogram LPJP1, which encodes NanoLuc luciferase, and successfully detected two subtypes of Listeria in just four hours [82]. In the same year, Yoshimitsu Masuda et al. introduced an LLB-producing phage (LLB-phage). Using genetic engineering to introduce the LLB structural gene into the lytic phage genome, this team successfully constructed a novel antimicrobial agent [83].

### 6.2. CRISPR-Cas System for Editing of Phage Genome

The recently discovered clustered regularly interspaced short palindromic repeats-associated protein (CRISPR-Cas) system is involved in the natural protection of prokaryotes from foreign DNA invasion [84,85,86]. CRISPR-Cas can be used to edit phage genomes, thereby conferring the advantage of specific production of the expected recombinant phages, with the removal of Cas protein inhibiting the production of wild-type phages. In recent years, the CRISPR-Cas system has been used for genome editing in several organisms, including phages. In general, the CRISPR-Cas system contains two important components: Cas protein and CRISPR RNA (crRNA). CRISPR-Cas for editing the phage genome involves three steps: (a) adaptation, (b) crRNA biogenesis, and (c) interference [87]. In the adaptation step, a short foreign nucleotide sequence (30–40 bp) called the “spacer or tracer” sequence is integrated with CRISPR loci of partially palindromic DNA repeats. In the crRNA biogenesis step, the spacer and partially palindromic DNA repeats are transcribed into crRNA. In the interference step, the crRNA combines with one or more CRISPR associated proteins (Cas) to form a complex called a “protospacer”, which will recognize the DNA and finally degrade it. Based on Cas protein function, CRISPR-Cas systems are classified into six types I to VI, with each of them having several subtypes based on genetic diversity. According to the current scenario, these six types belong to two major classes: the class 1 system encodes multiple Cas proteins involved in types I, III, and IV, and the class 2 system encodes a single Cas protein function to degrade target DNA in type II, V, and VI. In recent years, types I, II, and III CRISPR systems have been mainly used for phage genome editing [88]. The class 1 subtype I-E CRISPR-Cas system from *Vibrio cholerae* and *E. coli* and the class 2 subtype II-A system from *Streptococcus thermophilus* have been used to edit the genome of lytic phages. The CRISPR-Cas system was first used to delete gene 1.7 of T7 phage [89]. In the same year, the class 2 subtype II-A system from *S. thermophilus* was used for the first time [90]. In 2016, the *V. cholerae* phage was edited with a class 1 subtype I-E CRISPR-Cas system, which contains both donor DNA and the CRISPR-Cas gene, to successfully obtain recombinant phages [91]. The CRISPR-Cas9 protein system belongs to the type II CRISPR system, which requires a protospacer-adjacent motif (PAM) site to degrade foreign DNA [92,93,94]. The *Streptococcus pyogenes* Cas9 (SpCas9) protein has a special programmed PAM site containing three base pairs (5′-NRG-3′) to cleave target foreign DNA [95,96]. The sequence 5′-NRG-3, where N = any nucleotide A, T, G, or C, and R = G or A, presents a range of possible SpCas9 PAM sites: AGG, TGG, GGG, CGG, AAG, TAG, GAG, and CAG.

**Figure 10:**
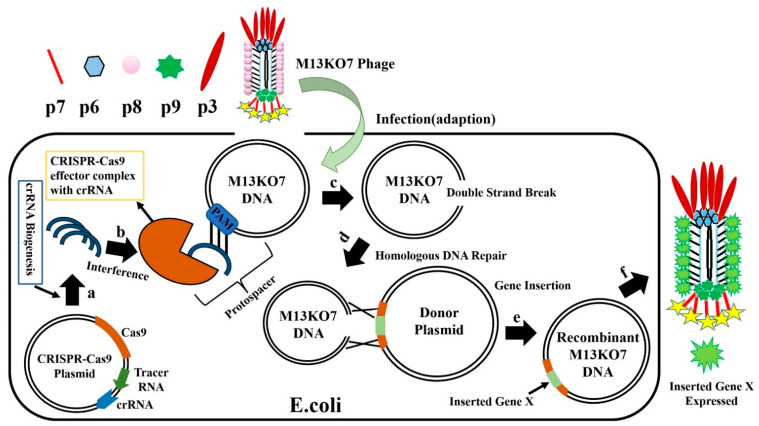
Overview of M13KO7 phage genome editing by CRISPR-Cas9. (**a**) The CRISPR-Cas9 plasmid synthesizes crRNA and CAS9 protein in *E. coli*, and forms the effector CAS9-crRNA complex; (**b**) *E. coli* carrying the CRISPR-Cas9-SgrNA plasmid is infected with phage M13KO7, and the CAS9-CrRNA complex binds to Phage M13KO7 DNA containing PAM sites; (**c**) Cas9-crRNA complex forms a double-strand break inM13KO7 phage DNA; (**d**) using homologous DNA repair, the donor plasmid carrying the novel gene repairs the broken M13KO7 phage DNA; (**e**) gene repair generates the recombinant M13KO7 phage-carrying gene X. (**f**) After translation and packaging, the recombinant M13KO7 phages-expressing gene X is secreted from *E. coli*.

SpCas9 targets and cleaves DNA with the help of the PAM site and spacer sequence already encoded in crRNA. The CRISPR-Cas system with SpCas9 has been used to edit several organisms, including phage genomes. When using this system, Cas9, crRNA, and tracr-RNA (trans-activating crRNA) were simultaneously cloned into the same plasmid [97,98,99]. When the crRNA and tracr-RNA are fused to generate a single guide RNA (sgRNA), the properly prepared highly active sgRNA yields positive results. After the *E. coli* with CRISPR-Cas9-sgRNA plasmid is infected with M13KO7 phage (Figure 10), the CRISPR-Cas9 plasmids synthesize crRNA and Cas9 protein, forming the effector Cas9-sgRNA complex. This complex has an ability to bind to the M13KO7 phage DNA at the PAM site and create a double-strand break. The broken M13KO7 phage DNA could then be repaired by the donor plasmid with Gene X in the middle of the short (40–60 bp) homologous sequence of gVIII (excluding PAM site) based on the DNA repair pathways of nonhomologous end joining or homologous DNA repair. After this repair pathway, gene insertion results in the production of the recombinant M13KO7 phage with the gene X peptide at the pVIII N-terminal. Unfortunately, a weak sgRNA may lead to false-positive results. The variable activity of crRNA has already been observed in Type I-E and Type IA CRISPR-Cas systems. Since the exact distribution of sgRNA activity in phages is still highly unknown, the prediction of high activity sgRNA in phages currently uses the sgRNA prediction tool based on a eukaryotic dataset [100,101,102]. Conventional recombination technology is highly expensive and time consuming for gene editing, which requires a few rounds of PCR, restriction enzyme digestion, and ligation [103]. These steps have been eliminated by the introduction of oligonucleotide recombination of the template through CRISPR-Cas systems, but the synthesis of long oligonucleotides still has limitations [104]. In 2018, the CRISPR-Cas system from *Listeria monocytogenes* was used for *Listeria* phage genome editing [105]. Later, the Type III CRISPR-Cas10 system was also developed and used for editing the staphylococcal phage genome [106]. The CRISPR-Cas10 system provides more protection to the host bacteria by exhibiting high cleavage activity toward the staphylococcal phage.

## 7. Challenges and Outlook of Phage-Based Biological Detection

As bacterial viruses, phages range in size from tens of nanometers to hundreds of nanometers; therefore, they can be regarded as biological nanomaterials. In addition, phages have evolved tail filaments or capsid proteins that are responsible for specifically attaching host bacteria. These characteristics of host recognition and small size enable phages to become highly efficient nanoprobes for capturing targets of interest. Because most phages can tolerate the insertion of foreign genes (20 bp to >1 kb), the common phages T7 (>1 kb) and M13 (<1500 bp) have been modified to construct phage probes for biological detection in recent years [107] (Deng et al., 2018). At present, homologous recombination and Crisp-Cas9 are the main techniques used for genetic modification of phages. With these two techniques, phages can be readily engineered into fluorescent or functionalized phage probes, which can be further modified with metal nanomaterials to develop organic–inorganic hybrid probes for the detection of various biological species. Although phages have been used for biological detection, there are some challenges in signal acquisition and effectiveness. For example, the M13 phage-based probe used for detection usually displays scFv fused with the P3 protein. The M13 phage is a filamentous phage with a length of 900 nm, and the P3 protein of M13 phage is a crucial site for the binding of M13 phage and foreign peptides and proteins. Once the p3 is disrupted, the infectivity of the M13 is lower, which was confirmed by titer experiment. Suppose the length of the phage is not shortened. In that case, the probe efficiency is very low due to the following reasons: (1) its long body would randomly intertwine in the solution, interfering with the p3 head of the probe binding to the target molecule; (2) since the head of M13 phage makes up a small proportion of the whole body (<2%), the probability of collision between the head and the target is very low. Therefore, the shortened phage probe will bind the target efficiently if the M13 phage can be reduced to 100 nm in length while retaining the p3 head used for target recognition. However, the mass production of shortened phages remains a challenge. Although some scholars have succeeded in producing a shortened M13 of 50 nm length, the rate of phage propagation is very low. According to the results of our repeated experiment, the titer of shortened M13 was only one-millionth that of the wild type cultured in host bacteria. The reason for this low titer remains unclear. In addition, other granular phage genetic modifications have been characterized, such as in T7 phage, whose capsid proteins can accommodate relatively large peptide fragments but only show a small number on their surfaces. Therefore, its sensitivity as a genetically modified fluorescent protein for fluorescence detection is limited. These problems necessitate further studies on phage packaging and display mechanisms for better application of phage-based probes.

In general, phage-based probes for biological detection are an emerging field of research that has demonstrated its capability in molecular diagnosis. For example, the composite probe assembled from T7 phage and GNPs can detect microRNA with a LOD of 3–5 aM, which exceeds the sensitivity of fluorescence quantitative PCR technology [36]. M13 phage-based nanoprobes can rapidly detect viruses with high sensitivity [38]. Although some scientific issues still need to be resolved before phage-based methods are widely accepted for clinical application, the target specificity of their amenability for genome editing and chemical modification differentiates phage-based detection methods from ELISA, PCR, and a variety of technologies based on synthetic polymer microspheres or metal nanoparticle probes. Thus, phage technology can be integrated with the multidisciplinary fields of nanotechnology and biotechnology for future application in various scenarios.

## Data Availability

Not applicable.
